# Some Incompatibilities

**Published:** 1895-12

**Authors:** J. S. Cassidy

**Affiliations:** Covington, Ky.


### Some Incompatibilities.

BY J. S. CASSIDY, COVINGTON, KY.

Fashions in the exhibition of medicines, as in other things, have their periods of change. This fact obtains in the selection of drugs for either local or internal use.

Thus, there are many physicians yet living who in their earlier years of practice gave calomel, *ad libitum*, to the point of mercurial saturation; it was the distinctive fad in those days, possibly because of a vague idea, an adumbration, as it were, of the present accepted theories of bacteriology, that big doses would be destructive to the materials involved in both the causes and consequences of the disease in question.

A few Samsons, as they were called, were regarded as the principal individual "bases" of various combinations, which also usually contained multitudes of auxiliaries and correctives that were pretty sure to "catch the coon either comin' or goin'."

In these combinations there naturally and unwittingly were too frequent association of decided incompatibles; and although it is still quite proper to make a judicious selection of effective drugs in a given formula, the strong tendency now-a-days is to write prescriptions that include but few ingredients.

Indeed, it is becoming more and more the style to order only one article, but of recognized utility, a habit due doubtless to the many pharmaceutical preparations which have become official, and which, although multiform in their composition, have been given each an individual name, thus simplifying matters very much; so much so that possibly even now, to say nothing of the immediate future, a paper of this sort may be somewhat out of place, and yet man is not and will not be perfect; if he were so, no suggestions would be needed, and no meetings of this kind would be held. Were it possible for George Eliot to have described the character of a man like Adam Bede, and at the time impress upon his mind the existence of the unconscious weakness of Hettie Sorrell, that pathetic story of the incompatibility in the moral nature of these two every-day examples of poor humanity would not have been written; neither would this paper have been conceived and inflicted thus upon you had I known in advance that all of us were still acquainted with the antagonistic qualities belonging to some of the drugs in common use.

I have known a patient who successively and repeatedly visited two specialists during the same half-hour; one of them would wash out the antrum and nares with solution of common salt—a good medicine—and then the other would effectually swab the throat and contiguous parts with solution of silver chloride. Another case somewhat similar—passive hemorrhage of the gums, treated locally by free application of tannin.

In a few hours another doctor applied perchloride of iron. Enough ink was manufactured by this process to convince doctor No. 2 that a good thing in the wrong place might be worse than a negative quantity. These two homely illustrations are in evidence against the evil tendency of too much subdivision in the field of practice; that although medical men, including dentists, are rapidly adopting mono-pharmacy, the specialists are increasing in number, and inasmuch as each specialist is a law unto himself in regard to his favorite medicines, there must be many cases of a separate and successive reception of incompatibles by the same unfortunate patient. It is well known that when two or more of the major number of soluble salts are mixed together in solution they are prone to exchange their radicals, and thus lose their former identity. Sometimes, however, they are given with this object in view, as, for instance, to obtain in a pleasant way the benefits of potassium citrate, potassium bicarbonate and hydrogen citrate are mixed, and the result secured.

There are, of course, exceptions to this rule of salts thus suffering mutual decomposition. Equal quantities of sodium chloride and potassium permanganate in aqueous solution produce an excellent disinfectant and antiseptic wash for either suppurating or simply inflamed surfaces; to these effects the practitioner might wish to add a stimulant; if alcohol, for instance, is the one chosen, it will violate the virtue of the others, and a dark magma, explosive when dry will be the paincipal issue of the union. The number of illegitimate births that may occur by union of a few apparently useful, if not harmless, bodies are illimitable. The characteristics of some of them are rather absurdly inconsistent in the light of their intended vocation, and others somewhat serious.

Potter says that "catechu potassium chlorate in a dentifrice have exploded in the mouth from the friction produced by a dry toothbrush." We all know of the colorless tincture of iodine developed by ammonia, which, instead of being what its name indicates in point of activity, ammonium iodide, a very dangerous substance. Electrozone, a new disinfectant produced by partial electrolysis of sea water, is incompatible with the same substances that antagonize Labarraque's solution, its chief active principle being sodium hypochlorate.

Analogous compounds of iodin and bromin are also present, and although in limited quantities, their virtues are not to be overlooked if we wish to receive the full benefits of this new candidate for professional favors, and, therefore, should be guarded against the many incompatibilities that necessarily affect the good influences of anything, medicinal or otherwise, which lays claim to Uncle Sam's motto "many in one." A number of authors could be quoted on this subject, and many instances selected from dental and medical journals to prove that the prescriber is too often carelessly forgetful of his duties in this respect; but I wish to make this paper brief, and to mention, merely in the way of a text, only a few examples of those incongruities which have come under my own observation. The fact is, since thinking over this matter, I am disposed to modify my indignation against a very dear friend, one of the most successful dentists of Cincinnati, for the statement he made in a paper read at a recent meeting of the Odontological Society of this city, "that the only medicines a dentists really needed, in order to prepare for a long and successful career in practice, are a barrel of carbolic acid and a pound of arsenic."

